# A Discontinuous Galerkin Model for Fluorescence Loss in Photobleaching

**DOI:** 10.1038/s41598-018-19159-7

**Published:** 2018-01-23

**Authors:** Christian V. Hansen, Hans J. Schroll, Daniel Wüstner

**Affiliations:** 1 Department of Mathematics and Computer Science, University of Southern Denmark, Campusvej 55, 5230 Odense M, Denmark; 2 Department of Biochemistry and Molecular Biology, University of Southern Denmark, Campusvej 55, 5230 Odense M, Denmark

## Abstract

Fluorescence loss in photobleaching (FLIP) is a modern microscopy method for visualization of transport processes in living cells. This paper presents the simulation of FLIP sequences based on a calibrated reaction–diffusion system defined on segmented cell images. By the use of a discontinuous Galerkin method, the computational complexity is drastically reduced compared to continuous Galerkin methods. Using this approach on green fluorescent protein (GFP), we can determine its intracellular diffusion constant, the strength of localized hindrance to diffusion as well as the permeability of the nuclear membrane for GFP passage, directly from the FLIP image series. Thus, we present for the first time, to our knowledge, a quantitative computational FLIP method for inferring several molecular transport parameters in parallel from FLIP image data acquired at commercial microscope systems.

## Introduction

Analysis of protein mobilities within living cells heavily relies on quantitative fluorescence microscopy. The protein of interest is either tagged with a green fluorescent protein (GFP) or its color variants. Alternatively, linkage tags are introduced genetically (as HaLo or SNAP tags) for subsequent labeling with suitable organic dyes^1–3^. The intracellular dynamics of such tagged proteins can be followed and quantified by three principal approaches (a) measurement of fluorescence fluctuations in the steady state, as in fluorescence correlation spectroscopy and its imaging variants^4,5^, (b) single molecule tracking (SMT) to gather an ensemble of trajectories of individual molecules^6,7^ and (c) local disturbance of the steady state by photobleaching followed by measurement of establishing a new steady state^2,8^. Here, we are concerned with the last approach only. The disturbance by localized photobleaching can be singular in time, as in fluorescence recovery after photobleaching (FRAP), continuous, as in continuous photobleaching (CP) or repeatedly pulsed, as in fluorescence loss in photobleaching (FLIP). In FRAP and CP, the fluorescence dynamics is typically only monitored at the site of bleaching^8,9^. Accordingly, only one temporal profile of fluorescence change is gathered in conventional FRAP and CP and can be used for subsequent modeling of binding and diffusion processes. This comes at the risk of parameter uncertainty and overfitting^8^, which is why more recent FRAP studies include the whole spatiotemporal profile involved in the bleach and recovery^10–14^. In FLIP, the whole cell is automatically monitored, i.e., inside and outside the bleached domain, thereby naturally providing a temporal fluorescence profile (i.e., fluorescence loss) at each pixel position. Thus, FLIP provides comprehensive quantitative data on fluorescence dynamics for the whole cell as a precondition for reliable data modeling. However, only a few attempts have been made so far, to infer transport parameters from FLIP image data^15–17^. Luedeke *et al*. used a compartment model in their FLIP data modeling, in which a Heaviside function was used to describe the FLIP cycle of bleaching and scanning^16^. This lead to a non-linear ordinary algebraic-differential equation system, which was solved numerically. Diffusion was not explicitly included in this model. Gruebele and colleagues (2014) performed numerical simulations of the underlying reaction-diffusion model, in which the reaction term described the localized bleaching process^15^. They discretized the whole cellular domain into a few subdomains and fitted the experimental fluorescence loss in each subdomain to several diffusion models. To include the complete spatiotemporal fluorescence loss profile, we presented previously a quantitative FLIP model using a pixel-by-pixel analysis with an empirical fitting function available as a plugin to the popular image analysis program ImageJ^17,18^. This analysis method allowed for detecting local heterogeneities in fluorescence loss kinetics, but the underlying causes could not be inferred from the empirical model used.

In^19^ we presented a reaction–diffusion compartment model for intracellular transport observed in FLIP images, which can describe both diffusion, nucleo-cytoplasmic transport, and local binding mechanistically. We focused on GFP, as many reference measurements by a variety of techniques are available, and because GFP is known to interact minimally, i.e., only non-specifically with intracellular structures^20–23^. Transport of GFP across the nuclear membrane is assumed to be passive, as the GFP sequence lacks nuclear import or export signals^21,24,25^. We modeled that process as a passive exchange of GFP between nucleus and cytoplasm by a stiff reaction. Resolving the rate coefficients and the strong gradient in intensity across the membrane by a continuous Galerkin method required fine meshes near and inside the membrane. Also, our previous model^19^ did not allow for parameter estimation, i.e., iterative refinement of the model parameter values given the data. This, however, is most wanted in using FLIP modeling as a quantitative tool for analysis of experimental image data. In contrast to^19^, in the present paper, we have improved upon previous work in the following respects: 1) the jump in intensity across the membrane is treated as discontinuity. A semipermeable membrane separates the nucleus from the cytoplasm, and transport across the membrane is established by an interface condition. The spatial resolution of the membrane is avoided by a discontinuous Galerkin (DG) method working on essentially smaller meshes. The DG–mesh for a typical FLIP image consists of 1500 triangles only in comparison to 221000 triangles in the continuous Galerkin simulation presented in^19^. Even though the DG method is about 5–20 times more computationally expensive than continuous Finite Elements^26^, one may expect up to 29 times faster execution for the DG simulation. 2) we have implemented a parameter estimation method, which allows us to directly determine diffusion constant, spatially varying binding constants and the nuclear membrane permeability. For that, we compare various optimization routines for iterative minimization of the error between FLIP image data and the DG model of the postulated underlying reaction–diffusion process. This is, to our knowledge, the first attempt of direct parameter inference from FLIP image data.

The outline of the paper is as follows: The next section presents a schematic description of the FLIP protocol. A reaction–diffusion PDE model is developed in Section 2. Incorporation of the membrane interface condition (Subsection 2.2) into the DG method is described in Section 3 while Section 4 comments on aspects of the implementation in the FEniCS project^27^. Calibrated simulations of FLIP sequences are presented in Section 5. A discussion including FLIP simulation of differently sized inert permeation probes combined with concluding remarks is provided in Section 6.

## Fluorescence Loss in Photobleaching

**Figure 1:**
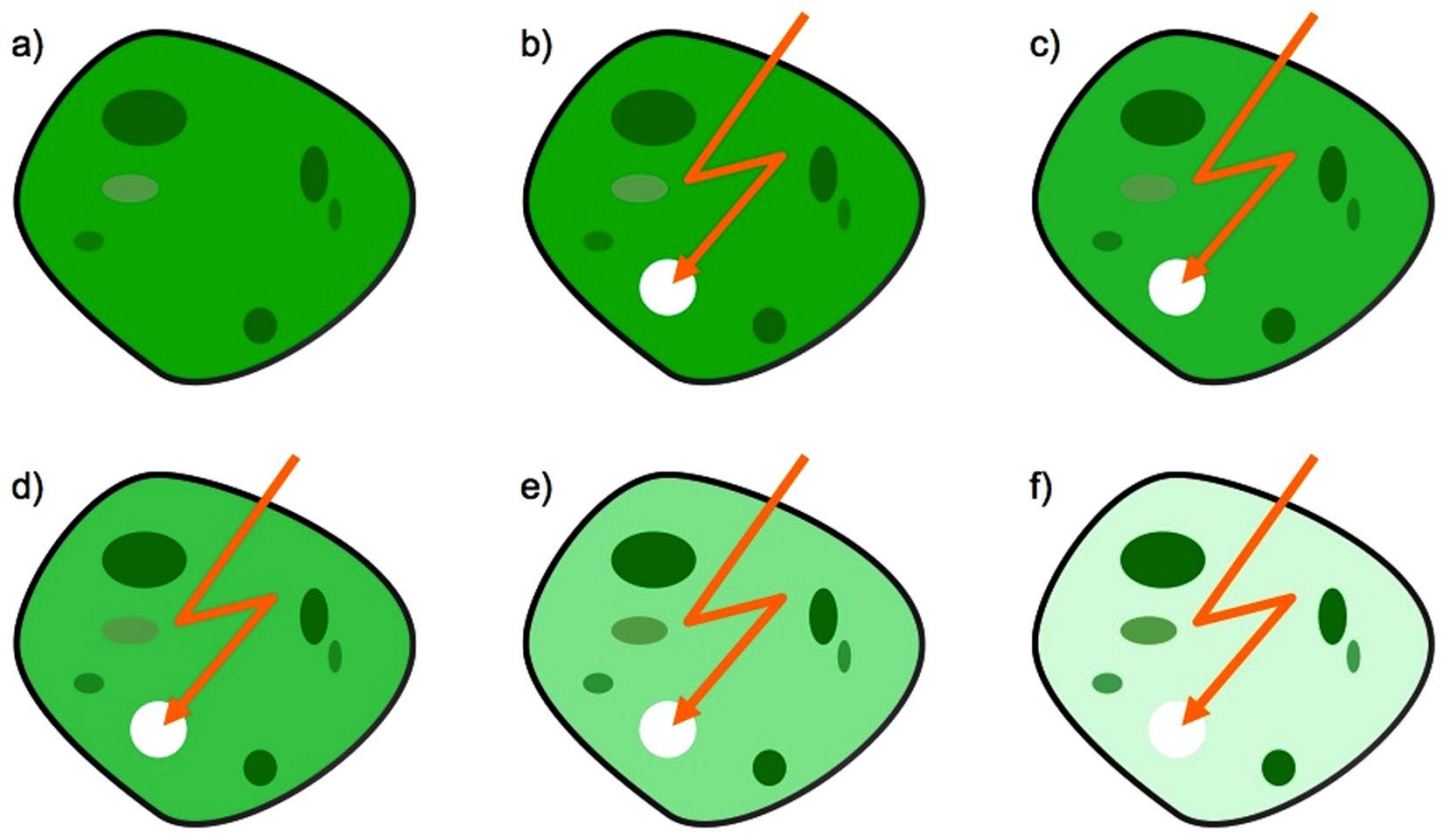
Schematic illustration of a FLIP experiment, for a cell containing green fluorescent molecules. (**a**) The cell at steady sate. (**b**–**f**) A small region is repeatedly bleached by laser light (orange flash). Fluorescence begins to fall in the cytoplasm and in some organelles (ellipses). This demonstrates diffusive transport of molecules towards the bleached region with recruitment from some of the organelles.

In FLIP a selected cell–area is repeatedly bleached using the intense laser beam of a confocal microscope. In between the bleaches, an image scan is made to observe the transport process, see Fig. 1 for illustration. The bleaching induces a decrease in fluorescence, not only in the bleaching area but in the whole cell due to the transport processes towards the repeatedly bleached area. This in principle allows evaluating the transport in the cell and between the intracellular compartments.

Thus, any delayed fluorescence loss in a particular cellular region outside the bleach spot indicates hindrance to molecular transport, either due to steric barriers to diffusion (for example the nuclear membrane separating cytosol from the nucleus), due to binding or because of crowding. The latter has been shown to cause excluded volume effects and, in the case of the nucleus, fractal diffusion as a consequence of the complex DNA folding and topology^17,20,28^.

## A reaction-diffusion model in segmented FLIP images

**Figure 2:**
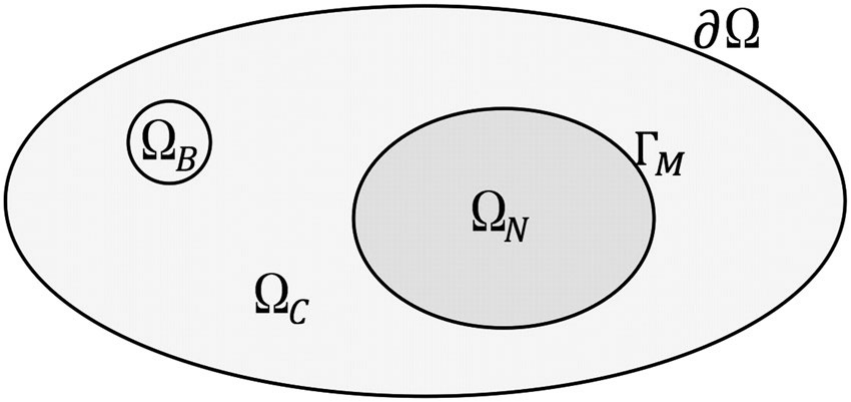
Schematic illustration of cell domains. The full cell domain is denoted Ω with boundary ∂Ω. Here ${\rm{\Omega }}={{\rm{\Omega }}}_{N}\cup {{\rm{\Omega }}}_{C}$, where Ω_*N*_ and Ω_*C*_ represent the nucleus and cytoplasm, respectively. Ω_*B*_ is the bleaching domain located in the cytoplasm, such that ${{\rm{\Omega }}}_{B}\subset {{\rm{\Omega }}}_{C}$. Γ_*M*_ is the boundary between Ω_*N*_ and Ω_*C*_, which represent the nuclear membrane.

The PDE model of the FLIP process is a reduced version of the system in^19^ defined on two compartments, namely nucleus and cytoplasm. To obtain a realistic simulation, the compartment boundaries are found via segmentation of the FLIP images. For this and later references, we will use Ω as a notation for the whole cell domain, and ∂Ω denotes the boundaries. Furthermore, we let Γ_*M*_ represent the nuclear membrane, Ω_*N*_ and Ω_*C*_ represent nucleus and cytoplasm respectively. In this paper we let the bleaching area be located within the cytoplasm Ω_*B*_ ⊂ Ω_*C*_, see Fig. 2.

The segmentation of the FLIP images is produced by the Chan-Vese active contours algorithm^29^. The algorithm is based on level set functions where the goal is to minimize the Chan-Vese energy functional by activating the level set function through an artificial time-like parameter. By minimizing the energy functional one minimizes the total deviation from the average gray-levels in for- and background, respectively. The energy functional also takes the length and thereby the smoothness of the curve into account. The implementation and further description of the Chan-Vese algorithm can be found in^19,30^. In this paper, the algorithm is applied to localize boundaries of the cell, nucleus and bleaching area in our FLIP images. Here the cell and bleaching area are segmented from the first FLIP image, while the nucleus is segmented from frame number 45, where the nucleus geometry is clearest.

### A reaction–diffusion model with hindrance

Inspecting the FLIP images, one of the most conspicuous things would be the architecture of especially the nucleus. There is currently put a lot of research effort on characterizing spatial heterogeneities in intracellular diffusion and transport processes. Especially within the nucleus, it is observed that molecular crowding hinder GFP’s diffusion in dense nuclear compartments^20,28^. GFP is considered as minimally interacting protein, such that specific binding to intracellular structures can likely be ignored. However, the spatial heterogeneity of GFP distribution, which we observed especially in the nucleus, indicates that the mesoscopic cellular organization together with non-specific interactions of eGFP can cause local enrichment or depletion of this protein. Such locally varying heterogeneous distribution of eGFP can be the consequence of protein partitioning into aqueous nuclear phases with differing properties^31^. Alternatively, it is the result of the fractal organization of diffusion barriers, for example stemming from the nuclear DNA content^20,28^. Such barriers to diffusion have been detected in the nucleus by pair correlation analysis of intensity fluctuations of eGFP^19^. Similarly, the heterochromatin-euchromatin border has been shown to form a barrier for protein diffusion^32^. In^17^ the pixel-wise FLIP analysis shows a negative correlation between DNA content and the fluorescence intensity and fluorescence loss kinetics of GFP in the nucleus. The computational FLIP model therefore needs to account for the uneven distributions of nuclear proteins.

We model the spatially varying eGFP distribution using rate constants and classical mass-action kinetics. It should be emphasized that this is a significant simplification, as diffusion of eGFP in the bounded state is ignored, and the underlying causes of local protein enrichment are not explicitly considered. However, as they are only partly understood, and we find good agreement of our simulation results with the experimental FLIP data, we use this pragmatic modeling approach here. More complicated modeling approaches including confined or anomalous diffusion will be discussed in section 6, below.

Thus the model consists of both hindered and free fluorescence proteins and we define the observed fluorescence intensity as:

1 $$c=u+{u}_{b},$$

where *u* and *u*_*b*_ is the intensities of the free and hindered molecules, respectively. The high-intensity areas are the areas in which we find that GFP is hindered in its motion. Thus, in these areas, *u* has been transformed into *u*_*b*_, in contrast to areas of low intensity. This is described by the reversible, first order reaction mechanism:

2 $${\rm{u}}\underset{{{\rm{k}}}^{-}}{\overset{{{\rm{k}}}^{+}}{\rightleftharpoons }}{{\rm{u}}}_{b},$$

where *k*^+^ and *k*^−^ are spatially resolved positive reaction constants; i.e. we account for the above mentioned diffusion barriers by a mean field approach using reaction rate constants *k*^+^ and *k*^−^.

Assuming diffusive transport of the free (but not the hindered) GFP–tagged molecules according to Fick’s law, the time-dependent PDE model reads:

$$\begin{array}{rcl}{u}_{t} & = & \nabla \cdot (\alpha \nabla u)+{k}^{-}{u}_{b}-{k}^{+}u-{\chi }_{{{\rm{\Omega }}}_{B}}\theta b\frac{q}{1+q}u,\\ {({u}_{b})}_{t} & = & {k}^{+}u-{k}^{-}{u}_{b}-{\chi }_{{{\rm{\Omega }}}_{B}}\theta b\frac{q}{1+q}{u}_{b},\quad {\bf{x}}\in {\rm{\Omega }},\quad t > \mathrm{0,}\end{array}$$

where *α* is the diffusion coefficient for free GFP molecules, *b* is the intrinsic bleaching rate constant, *q* is the equilibrium constant for the reaction between the ground and excited state for a fluorophore^33^, thus $b\tfrac{q}{1+q}$ is the total rate at which the fluorophores are bleached inside the bleaching area Ω_*B*_. Further, *θ* and ${\chi }_{{{\rm{\Omega }}}_{B}}$ are both characteristic functions, *θ* is time-dependent and simulates when the high-intensity laser bleaches, ${\chi }_{{{\rm{\Omega }}}_{B}}$ is space dependent and ensures that bleaching only occurs in the bleaching area:

$${\chi }_{{{\rm{\Omega }}}_{B}}=\{\begin{array}{cc}1 & {\rm{i}}{\rm{f}}\,{\bf{x}}\in {{\rm{\Omega }}}_{B},\\ 0 & {\rm{e}}{\rm{l}}{\rm{s}}{\rm{e}}.\end{array}$$

At the initial time, before bleaching, the system is in equilibrium and the free molecules are uniformly distributed *u*^0^ = const. Any higher fluorescence intensity is due to accumulation of hindered molecules ${c}^{0}({\bf{x}})={u}^{0}+{u}_{b}^{0}({\bf{x}})\ge {u}^{0}$. Thus, the initial intensity of free molecules is the uniform background of the observed initial intensity

3 $${u}^{0}=\mathop{min}\limits_{{\bf{x}}\in {\rm{\Omega }}}\,{c}^{0}({\bf{x}}).$$

The equilibrium state for (2) is given by ${u}^{0}{k}^{+}={k}^{-}{u}_{b}^{0}({\bf{x}})={k}^{-}({c}^{0}({\bf{x}})-{u}^{0})$. It is reasonable to model *k*^+^ = *k*^+^ (**x**) to be positive where increased fluorescence intensity indicates the presence of hindered molecules

4 $${k}^{+}({\bf{x}})=\gamma {u}_{b}^{0}({\bf{x}})=\gamma ({c}^{0}({\bf{x}})-{u}^{0}),$$

and *γ* is a proportionality constant. Consequently, *k*^−^ is constant

5 $${k}^{-}=\frac{{k}^{+}({\bf{x}})}{{u}_{b}^{0}({\bf{x}})}{u}^{0}=\gamma {u}^{0}.$$

### Compartment model with semipermeable membrane

To obtain a realistic FLIP simulation at least two compartments are needed, i.e., the cytoplasm and nucleus. These compartments are separated by the nuclear membrane. According to Fick’s first law the diffusive flux is anti-proportional to the gradient ${\bf{J}}=-\alpha \nabla u$. To model diffusive transport across a semipermeable membrane interface where *u* may jump, we integrate Fick’s law across the membrane to obtain ${\bf{J}}=-p({u}^{+}-{u}^{-}){{\bf{n}}}^{-}$. Here, *p* denotes the solute permeability of the membrane measured in *μm*/*s*. The membrane separates the domain into two compartments labeled by ± superscripts. In our cell model see Fig. 2 for example, the nucleus Ω_*N*_ is the minus-compartment and the cytoplasm Ω_*C*_ is the plus-compartment. The outward unit normal vectors *n*^±^ along the common interface point into the opposite compartment. If the concentration outside is greater than inside *u*^+^ > *u*^−^, then the flux points back into the minus-compartment resulting in a damping effect in agreement with Fick’s law. As the outward normals along a common interface are opposite, the flux may be written as a jump bracket

6 $${\bf{J}}=p({u}^{-}-{u}^{+}){{\bf{n}}}^{-}=p[\kern-2pt[ u]\kern-2pt] .$$

Despite the fact that biological transport across a membrane may be complex, it is common practice to approximate the permeability experimentally by dividing the measured flux by the jump in concentration^34,35^. At this point, we are ready to summarize the mathematical model.

### The complete PDE model

The fluorescence intensities of both free and hindered molecules are governed by the reaction diffusion system

7 $$\begin{array}{rcl}{u}_{t} & = & \nabla \cdot (\alpha \nabla u)+{k}^{-}{u}_{b}-{k}^{+}u-{\chi }_{{{\rm{\Omega }}}_{B}}\theta b\frac{q}{1+q}u,\\ {({u}_{b})}_{t} & = & {k}^{+}u-{k}^{-}{u}_{b}-{\chi }_{{{\rm{\Omega }}}_{B}}\theta b\frac{q}{1+q}{u}_{b},\quad {\bf{x}}\in {\rm{\Omega }},\quad t > 0.\end{array}$$

The reaction rates are taken from (4) and (5). Along the membrane, the diffusive flux (6) is expressed as interface condition

8 $${\bf{J}}\cdot {{\bf{n}}}^{-}=-\alpha \frac{\partial {u}^{-}}{\partial {{\bf{n}}}^{-}}=p[\kern-2pt[ u]\kern-2pt] \cdot {{\bf{n}}}^{-}\quad {\bf{x}}\in {{\rm{\Gamma }}}_{M}.$$

Focusing on the intracellular architecture and diffusive transport of GFP, we may assume there is no transport of GFP across the cell membrane ∂Ω

9 $${\bf{n}}\cdot \nabla u={\bf{n}}\cdot \nabla {u}_{b}=\mathrm{0,}\quad {\bf{x}}\in \partial {\rm{\Omega }}.$$

The normalised initial intensity 0 ≤ *c*(0, **x**) ≤ 1 is extracted from the first FLIP image and

10 $$u\mathrm{(0,}{\bf{x}})={u}^{0}=\mathop{{\rm{\min }}}\limits_{{\bf{x}}\in {\rm{\Omega }}}\,c\mathrm{(0},{\bf{x}}),\quad {u}_{b}\mathrm{(0},{\bf{x}})=c\mathrm{(0},{\bf{x}})-{u}^{0},\quad {\bf{x}}\in {\rm{\Omega }}.$$

## A discontinous Galerkin method with internal interface condition

To effectively simulate the abrupt change in fluorescence intensity as seen in FLIP images, it is desirable that the numerical method can represent discontinuous functions. The Discontinuous Galerkin (DG) method was first introduced by Reed and Hill^36^ in 1973 to resolve shocks in hyperbolic conservation laws. Independently, Babuska^37^, Wheeler^38^ and Arnold^39^ developed interior penalty discontinuous Galerkin (IPDG) methods for elliptic and parabolic problems. Since then the interest and the development of DG methods have been growing. The interested reader is referred to^40^ where the history of their development until 1999 can be found.

In this paper, the interface condition along the nuclear membrane (8) is implemented into the IPDG method based on^39,41^.

To describe the method, we introduce some notation. Let ${{\mathscr{T}}}_{h}$ denote the discretization of Ω into disjoint open elements ${\mathscr{K}}\in {{\mathscr{T}}}_{h}$. In connection, let Γ denote the union of the boundaries of all ${\mathscr{K}}$. Note that the mesh should be constructed such that ${{\rm{\Gamma }}}_{M}\subset {\rm{\Gamma }}$. Further we decompose Γ into three disjoint subsets ${\rm{\Gamma }}=\partial {\rm{\Omega }}\cup {{\rm{\Gamma }}}_{{\rm{int}}}\cup {{\rm{\Gamma }}}_{M}$, where Γ_int_ holds all internal edges ${{\rm{\Gamma }}}_{{\rm{int}}}:={\rm{\Gamma }}\backslash (\partial {\rm{\Omega }}\cup {{\rm{\Gamma }}}_{M})$. Further, let *u*^+^ and *u*^−^ denote a single valued function on two adjacent elements ${{\mathscr{K}}}^{+}$ and ${{\mathscr{K}}}^{-}$. As usual, **n**^±^ denote the outward unit vectors on along $\partial {{\mathscr{K}}}^{\pm }$. Then average and jump term are defined as {*u*} = (*u*^+^ + *u*^−^)/2, $[\kern-2pt[ u]\kern-2pt] ={u}^{+}{{\bf{n}}}^{+}+{u}^{-}{{\bf{n}}}^{-}$. For piecewise defined vector valued functions **q** let {**q**} = (**q**^+^ + **q**^−^)/2, $[\kern-2pt[ {\bf{q}}]\kern-2pt] ={{\bf{q}}}^{+}\cdot {{\bf{n}}}^{+}+{{\bf{q}}}^{-}\cdot {{\bf{n}}}^{-}$. Note that the jump of a scalar gives a vector, while the jump of a vector is a scalar, moreover

11 $$[\kern-2pt[ {\bf{q}}u]\kern-2pt] =[\kern-2pt[ {\bf{q}}]\kern-2pt] \{u\}+\{{\bf{q}}\}\cdot [\kern-2pt[ u]\kern-2pt] .$$

Consider the div–grad operator $\nabla \cdot (\alpha \nabla u)$ on two adjacent elements ${{\mathscr{K}}}^{\pm }$. By partial integration (Green’s first identity)

$${\int }_{{{\mathscr{K}}}^{\pm }}\,\nabla \cdot (\alpha \nabla u)v\,{\rm{d}}x=-{\int }_{{{\mathscr{K}}}^{\pm }}\,\alpha \nabla u\cdot \nabla v\,{\rm{d}}x+{\int }_{\partial {{\mathscr{K}}}^{\pm }}\,\alpha \nabla u\cdot {{\bf{n}}}^{\pm }v\,{\rm{d}}s,$$

where *v* denotes a suitable test function. Along the common edge $e=\partial {{\mathscr{K}}}^{+}\cap \partial {{\mathscr{K}}}^{-}$, normal derivatives sum up to a jump

$${\int }_{e}\,({\alpha }^{+}\nabla {u}^{+}{v}^{+}-{\alpha }^{-}\nabla {u}^{-}{v}^{-}){{\bf{n}}}^{+}\,{\rm{d}}s={\int }_{e}\,[\kern-2pt[ \alpha \nabla uv]\kern-2pt] \,{\rm{d}}s.$$

Summing up over all elements ${\mathscr{K}}\in {{\mathscr{T}}}_{h}$ we thus find

$${\int }_{{\rm{\Omega }}}\,\nabla \cdot (\alpha \nabla u)v\,{\rm{d}}x=-{\int }_{{\rm{\Omega }}}\,\alpha \nabla u\cdot \nabla v\,{\rm{d}}x+{\int }_{{{\rm{\Gamma }}}_{{\rm{int}}}}\,[\kern-2pt[ \alpha \nabla uv]\kern-2pt] \,{\rm{d}}s-{\int }_{{{\rm{\Gamma }}}_{M}}\,p[\kern-2pt[ u]\kern-2pt] \cdot [\kern-2pt[ v]\kern-2pt] \,{\rm{d}}s,$$

where both the membrane flux condition (6) and the zero flux boundary condition (9) have been used. The IPDG method enforces continuity across internal edges by a penalty term^37,39,41^. Using (11) the formula

$${\int }_{{{\rm{\Gamma }}}_{{\rm{int}}}}\,[\kern-2pt[ {\bf{J}}(u)v]\kern-2pt] \,{\rm{d}}s={\int }_{{{\rm{\Gamma }}}_{{\rm{int}}}}\,\{{\bf{J}}(u)\}\cdot [\kern-2pt[ v]\kern-2pt] \,{\rm{d}}s+{\int }_{{{\rm{\Gamma }}}_{{\rm{int}}}}\,\{{\bf{J}}(v)\}\cdot [\kern-2pt[ u]\kern-2pt] \,{\rm{d}}s-{\int }_{{{\rm{\Gamma }}}_{{\rm{int}}}}\,\frac{\sigma }{h}[\kern-2pt[ u]\kern-2pt] \cdot [\kern-2pt[ v]\kern-2pt] \,{\rm{d}}s$$

is symmetric and consistent for continuous solutions $[\kern-2pt[ u]\kern-2pt] =[\kern-2pt[ {\bf{J}}(u)]\kern-2pt] =0$. Here *h* denotes the average diameter of two adjacent elements, and *σ* is the Nitsche parameter^42^.

The bilinear form for the div–grad operator based on the IPDG method reads

12 $$D(u,v,\alpha ):={\int }_{{\rm{\Omega }}}\,\alpha \nabla u\cdot \nabla v\,{\rm{d}}x-{\int }_{{{\rm{\Gamma }}}_{{\rm{int}}}}\,\{\alpha \nabla v\}\cdot [\kern-2pt[ u]\kern-2pt] \,{\rm{d}}s-{\int }_{{{\rm{\Gamma }}}_{{\rm{int}}}}\,\{\alpha \nabla u\}\cdot [\kern-2pt[ v]\kern-2pt] \,{\rm{d}}s+{\int }_{{{\rm{\Gamma }}}_{{\rm{int}}}}\,\frac{\sigma }{h}[\kern-2pt[ u]\kern-2pt] \cdot [\kern-2pt[ v]\kern-2pt] \,{\rm{d}}s.$$

The last integral in (12) is the internal penalty; a large enough Nitsche parameter enforces continuity across internal edges^37,39,41,42^. Let *v* and *w* be discontinuous, piecewise bilinear test-functions for *u* and *u*_*b*_ respectively. The semi–discrete PDE with boundary condition and interface conditions (8) and (9) reads

13 $$\begin{array}{rcl}{\int }_{{\rm{\Omega }}}\,{u}_{t}v\,{\rm{d}}x+D(u,v,\alpha ) & = & R(u,{u}_{b},v)-B(u,v)-p{\int }_{{{\rm{\Gamma }}}_{{\rm{M}}}}\,[\kern-2pt[ u]\kern-2pt] \cdot [\kern-2pt[ v]\kern-2pt] \,{\rm{d}}s,\\ {\int }_{{\rm{\Omega }}}\,{({u}_{b})}_{t}w\,{\rm{d}}x & = & -R(u,{u}_{b},w)-B({u}_{b},w),\end{array}$$

with $$R(u,{u}_{b},v)\,:={\int }_{{\rm{\Omega }}}\,({k}^{-}{u}_{b}-{k}^{+}u)v{\rm{d}}x$$ and $$B(u,v)\,:={\int }_{{{\rm{\Omega }}}_{B}}\,\theta b\frac{q}{1+q}uv{\rm{d}}x$$.

We discretize the time derivative by a backward Euler step. Any higher order but L-stable method, like certain SDIRK schemes^43^, are appropriate as well.

## FEniCS implementation

Applying a backward Euler time step to (13) results in the weak form for the time step

$$\begin{array}{rcl}{\int }_{{\rm{\Omega }}}\,\frac{{u}^{n+1}-{u}^{n}}{{\rm{\Delta }}t}v\,{\rm{d}}x+D({u}^{n+1},v,\alpha ) & = & R({u}^{n+1},{u}_{b}^{n+1},v)-B({u}^{n+1},v)-p{\int }_{{{\rm{\Gamma }}}_{M}}[\kern-2pt[ {u}^{n+1}]\kern-2pt] \cdot [\kern-2pt[ v]\kern-2pt] \,{\rm{d}}s,\\ {\int }_{{\rm{\Omega }}}\,\frac{{u}_{b}^{n+1}-{u}_{b}^{n}}{{\rm{\Delta }}t}w\,{\rm{d}}x & = & -R({u}^{n+1},{u}_{b}^{n+1},w)-B({u}_{b}^{n+1},w\mathrm{)}.\end{array}$$

This weak form is conveniently implemented using the automated Finite Element package FEniCS^27^. For faster execution, it is recommended to pre–assemble the system matrix, which FEniCS can do automatically based on the given mesh and weak formulation. The pulsating laser is realized by pre–assembling two systems, with and without the bleaching term *B*(*u*, *v*). To resolve the effect of bleaching, the bleaching interval is a multiple of the time step: Δ*t*_*b*_ = *m*Δ*t*, $m\in {\mathbb{N}}$. To use FEniCS a high-level Python script is written, where the weak formulation is expressed in the UFL form language. UFL is a domain specific language for defining weak formulations in a notation close to the one presented in this paper^44^. DOLFIN then interprets the script and passes the UFL to the Variational Form Compiler (FFC). Then Instant (build on top of SWIG) turns it into a C++ function callable from Python. In the end, the linear systems are solved by the UMFPACK sparse, direct solver via PETSc^45,46^. Optional iterative and parallel solvers are available. A test on the given mesh and the system from this paper showed that the iterative generalized minimal residual method with PETSc algebraic multigrid preconditioner was overall 20–30% slower than the direct solver.

## Calibration and simulation of FLIP images

**Figure 3:**
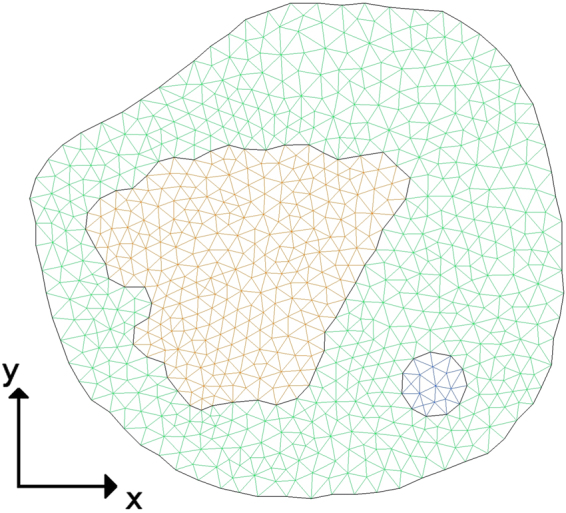
Finite element mesh on Chan-Vese active contours generated by Gmsh. Green: cytoplasm, orange: nucleus, blue: bleaching area.

The discontinuous Galerkin method approximates the solution to the PDE model (7)–(10) as a piecewise bilinear and possibly discontinuous function defined on a triangulation of the cell. The discrete geometry from the segmented FLIP images is written into a .geo geometry file. By Gmsh^47^ the mesh is constructed on the segmented cell geometry found in the geo file and displayed in Fig. 3. It consists of 1523 triangles; 991 located in the cytoplasm, 503 in the nucleus and 29 in the bleaching area.

The initial fluorescence intensity 0 ≤ *c*(0, **x**) ≤ 1 is extracted from the first FLIP image. The original images are affected by some noise, however. Therefore, the FLIP images are preconditioned by Gaussian smoothing (with a radius of one pixel = 0.05467326 *μ*m) within the cell domain. The intensity of free and hindered molecules is initialized according to (10) i.e., the intensity pattern as seen in the first blurred FLIP image is carried by the hindered molecules.

The bleaching time interval was Δ*t*_*b*_ = 0.8 s followed by a recovery phase of 1.8 s, resulting in a total frame rate of 2.6 s. For the simulation the discrete time step is set to be Δ*t* = 0.2 s.

Not yet defined model parameters are: the diffusion coefficient *α*, the bleaching term $\beta =b\frac{q}{1+q}$ both appearing in the PDE model (7), the proportionality factor *γ* in reaction rates (4) and (5), as well as the permeability constant *p* in the interface condition (8).

### Calibration

The remaining parameters are identified by calibrating the simulation to observed FLIP images. To this end, a misfit functional is minimized with respect to the parameters. At discrete times *t*_*i*_ = 2.6(*i* − 1) + 2.0 seconds *i* = 1, 2, 3, …, 50 we measure the difference between the simulated intensity and the preconditioned (blurred) FLIP images represented as a piecewise linear finite element function on the mesh. For tests regarding the number of FLIP images used, see Supplementary S.3. Thus, the misfit functional is expressed as

14 $$E=\frac{1}{50}\,\sum _{i=1}^{50}\,{\int }_{{\rm{\Omega }}}\,{|u({t}_{i},{\bf{x}})+{u}_{b}({t}_{i},{\bf{x}})-{c}_{g}({t}_{i},{\bf{x}})|}^{2}\,{\rm{d}}x,$$

where *c*_*g*_ denotes the intensity of the goal function. Squaring the deviation puts a strong penalty on outliers and results in a more even distribution of residuals. The PDE constrained calibration problem reads:

$$(\bar{\alpha },\bar{\beta },\bar{\gamma },\bar{p})=\mathop{{\rm{argmin}}}\limits_{0 < \alpha ,\beta ,\gamma ,p}\,E(\alpha ,\beta ,\gamma ,p),$$

where *u* and *u*_*b*_ solve the PDE model (7). To perform the optimization, we apply the Nelder–Mead downhill simplex algorithm^48^ which is part of the SciPy library^49^. It calls the semipermeable membrane FLIP model (13) implemented as a FEniCS function. Initially the Nelder–Mead search constructs with five initial guess vectors *ξ*_*k*_ = (*α*_*k*_, *β*_*k*_, *γ*_*k*_, *p*_*k*_) forming a four dimensional simplex. The misfit functional (14) is evaluated in all five vertices *E*_*k*_ = *E*(*ξ*_*k*_) and the vortices are renumbered in ascending order ${E}_{1} < {E}_{2} < \cdots  < {E}_{5}$. The least optimal simplex vector *ξ*_5_ is replaced by a (hopefully) better approximation. The iteration stops if both the progress in the optimal parameters $\Vert {\xi }_{1}^{(n+\mathrm{1)}}-{\xi }_{1}^{(n)}\Vert$ and the variation of the misfit functional *E*_5_ − *E*_1_ are small. Default tolerances are 10^−4^. For details, we refer to^48–50^. The performance of alternative algorithms and norms is discussed in Supplementary S.4 and S.5, respectively.

**Figure 4:**
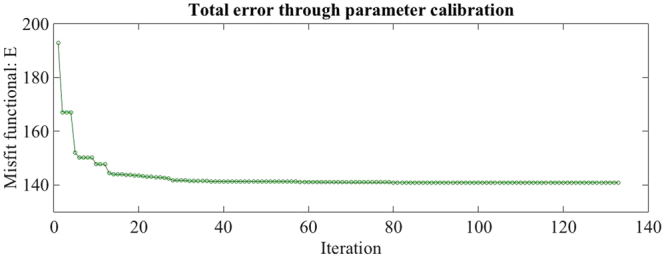
Plot of error *E* during the optimization process for the semipermeable membrane model.

**Figure 5:**
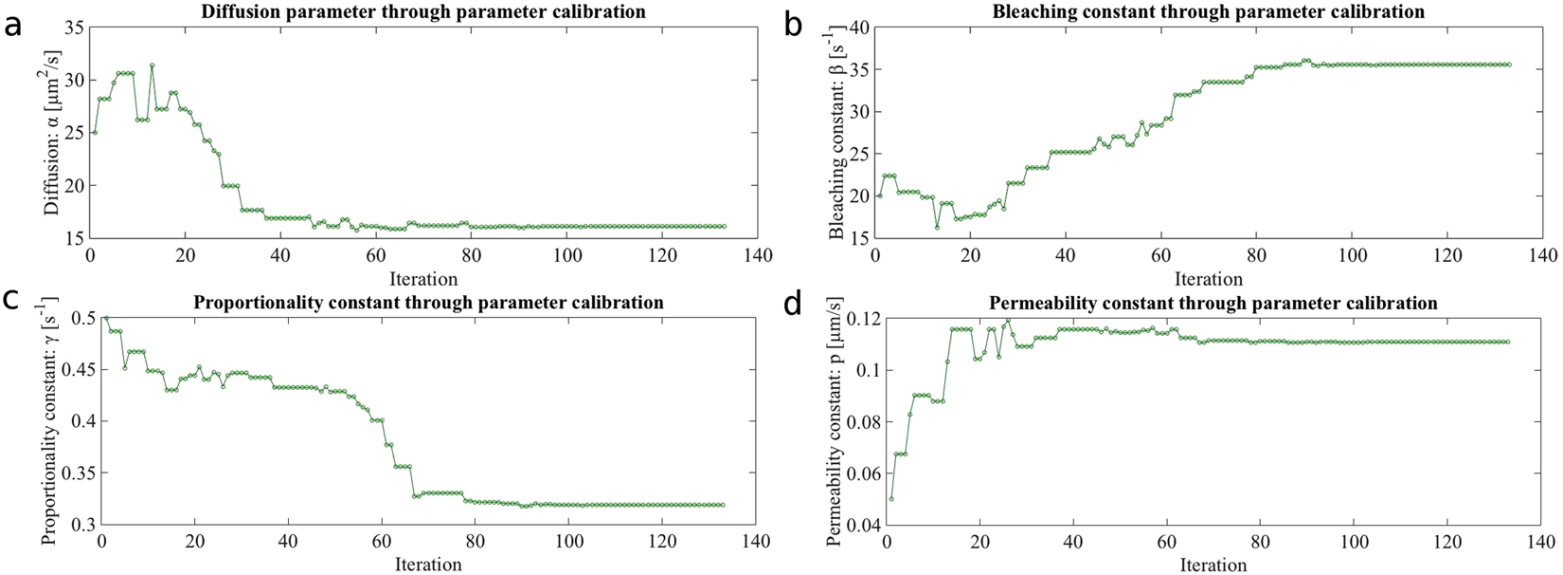
Parameters during the optimization process for the semipermeable membrane model.

Based on the literature^17,51^ and numerical experiments in^19^ a qualified initial guess is: *α*_0_ = 25, *β*_0_ = 20, *γ*_0_ = 0.5 and *p*_0_ = 0.05. Figure 4 depicts the progress of the optimization process with corresponding parameters shown in Fig. 5. We clearly observe the monotone decrease of the misfit functional from initially *E* = 193 down to *E* = 140 after 133 iterations with totally 231 function evaluations. The estimated parameters are

15 $$\bar{\alpha }=16.1,\quad \bar{\beta }=35.6,\quad \bar{\gamma }=0.319,\quad {\rm{and}}\quad \bar{p}=0.111.$$

The Nelder–Mead algorithm can call the FLIP solver multiple times per iteration, here resulting in 231 function evaluations in form of forward solutions of the PDE system (7)–(10). The calibration process takes approximately 3 hours on an Intel Core i5 processor at 3.2 GHz with 8 GB memory running Ubuntu 14.04.5.

### Simulation and visualisation

**Figure 6:**
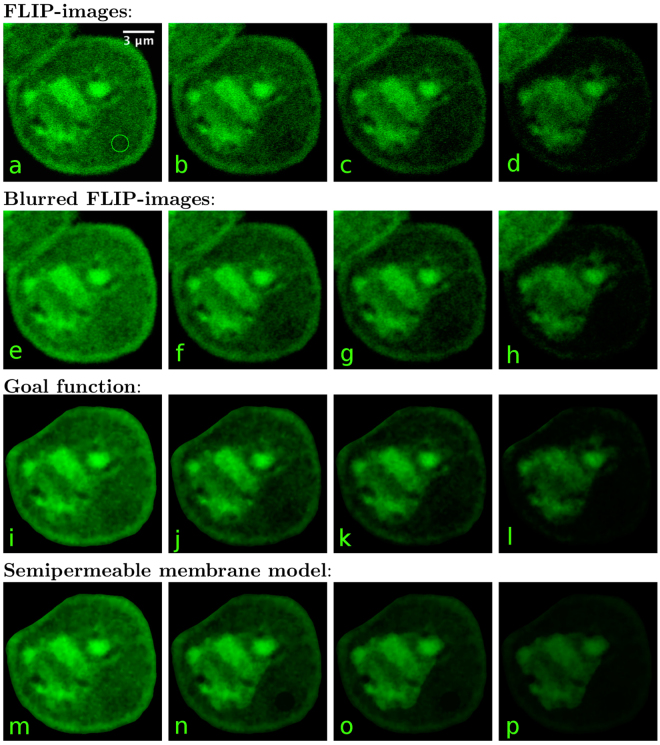
The first four images (**a**–**d**) are the original FLIP images of the McArdle RH7777 cells expressing GFP in the cytoplasm and nucleus. The green circle on the image (**a**) shows the 30-pixel wide bleaching area. The left most FLIP image (**a**) is taken before bleaching, the next image (**b**) is taken after it has been bleached 10 times i.e. time *t* = 26 s. The third FLIP image (**c**) is the 20’th FLIP image in the sequence (time *t* = 52 s) and the last (**d**) is at time *t* = 104 s which correspond to FLIP fame 40. The second row (**e**–**h**) shows the corresponding Gaussian blurred (radius = 1 px) FLIP images. The third row (**i**–**l**) shows the goal function and the last row (**m**–**p**) shows the simulation results, all at times corresponding to the displayed FLIP images.

With the optimized parameters (15) our FLIP model as stated in Section 2.3 is completely determined. Recall that reaction rates *k*^±^ as well as initial intensities are extracted from the first (denoised) FLIP image. A sequence of FLIP images in McArdle RH7777 cells is displayed in the top row of Fig. 6(a–d). Green fluorescent protein (GFP) was repeatedly bleached with full laser power at a 30 pixel (1.64 *μ*m) diameter circular region in the cytoplasm (green circle), in a temperature controlled (35 ± 1 °C) environment of a Zeiss LSM 510 confocal microscope using the 488-nm line of an Argon laser. The entire images were scanned with 0.5% laser power between each bleach. The total frame rate inclusive bleaching was 2.6 s and the image area is approximately 15 × 15 *μ*m. As mentioned earlier, we use Gaussian blur with radius 1 pixel to denoise the FLIP image. The blurred FLIP sequence is presented in the second row of Fig. 6(e–h). The first blurred FLIP image is used to create *k*^+^ and the subsequent is used to generate goal functions. A goal function is a piecewise linear discontinuous Galerkin function defined on the mesh, based on the pixel values from the blurred FLIP images. The goal functions displayed in the third row of Fig. 6(i–l) were used to calibrate the FLIP model. Finally, the simulation results of our calibrated FLIP model can be seen in the lowest row of Fig. 6(m–p).

**Figure 7:**
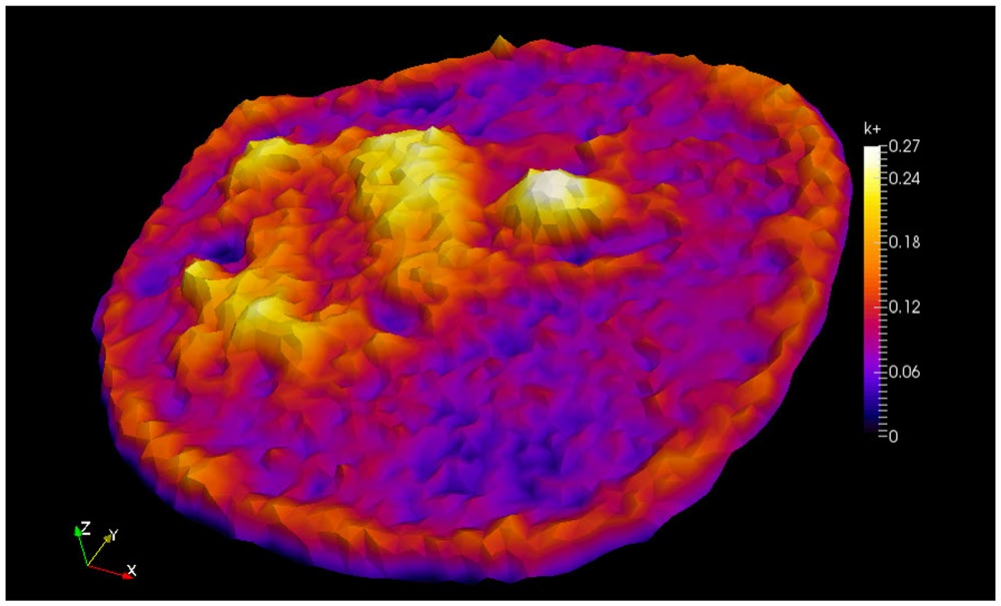
Plot of the reaction coefficient *k*^+^ with *γ* = 0.319. Note that in areas where a low number of hindered molecules are observed on the FLIP images *k*^+^ is low and in areas with high intensity *k*^+^ is also high.

The structure established in the simulation mainly originates from the reaction kinetics given in (2). In Fig. 7 *k*^+^ is illustrated based on the estimated proportional factor $\bar{\gamma }=0.319$. One can clearly see that the spatial map of *k*^+^ resembles the structure from the first intensity image as stated in (4). Hindrance to free diffusion is clearly higher in the nucleus compared to the cytoplasm, which is in accordance with earlier studies^10,20^.

## Discussion and Conclusion

To compare the spatiotemporal profile of fluorescence loss between experiment and simulation, we make use of our previously developed method, namely to fit a stretched/compressed exponential (StrExp) function to each pixel position in the data and simulation outputs^17^. This function is an extension of the exponential function, as it can be considered as the sum of exponentials with a distribution of rate constants, rather than a single rate constant. This leads to a time-dependent rate coefficient, suitable for modeling delays and long-tail kinetics, not addressable using a single exponential decay function. The StrExp function is widely used for modeling physico-chemical processes and is used here to provide an independent assessment of the quality of our FLIP model. The StrExp function provides an accurate description of fluorescence loss kinetics and reads with amplitude map *I*_0_(**x**), time constant map, *τ*(**x**), heterogeneity map, *h*(**x**) and a background term, *I*_*b*_(**x**)^17,52–54^

$$I({\bf{x}},t)={I}_{0}({\bf{x}})\,\exp \,[-{(\frac{t}{\tau ({\bf{x}})})}^{\frac{1}{h({\bf{x}})}}]+{I}_{b}({\bf{x}}).$$

The heterogeneity parameter describes the shape of the intensity decay with 0 ≤ *h* < 1 modeling a delayed (compressed) exponential and 1 < *h* ≤ 2 modeling a stretched exponential, which is faster than exponential initially and slower for long times compared to *τ*. For *h* = 1, one recovers a mono-exponential function. We showed previously, that the StrExp function can accurately model diffusional transport in FLIP simulations, both in 2D and in 3D. We found that the shape of the fluorescence loss profile is well approximated with 1 < *h* ≤ 2 inside the bleach spot and a gradient of h-values as function of distance from the bleaching spot in the range 0.5 ≤ *h* < 1 outside the bleached region^17^. We demonstrated also that binding/release-dominated transport can be fitted with a StrExp function as well. Finally, we found that local heterogeneity in the h-map between neighboring pixels for GFP FLIP experiments indicates deviation from classical diffusional transport with space-invariant diffusion constant in living cells. In fact, we found for exactly the same experimental FLIP sequence used in the current study, that pixel-to-pixel variation of h-values, either larger or smaller than one exist in the cytoplasm and in the nucleus (see Figs 4 and 5 in^17^). This can be seen particularly clearly when calculating the rate coefficient map, which is defined as:

16 $$k({\bf{x}},t)=-\frac{{\rm{\partial }}\,{\rm{l}}{\rm{n}}\,{I}_{n}({\bf{x}},t)}{{\rm{\partial }}t}=\frac{1}{h({\bf{x}})\tau ({\bf{x}})}{(\frac{t}{\tau ({\bf{x}})})}^{\frac{1}{h({\bf{x}})}-1}$$

**Figure 8:**
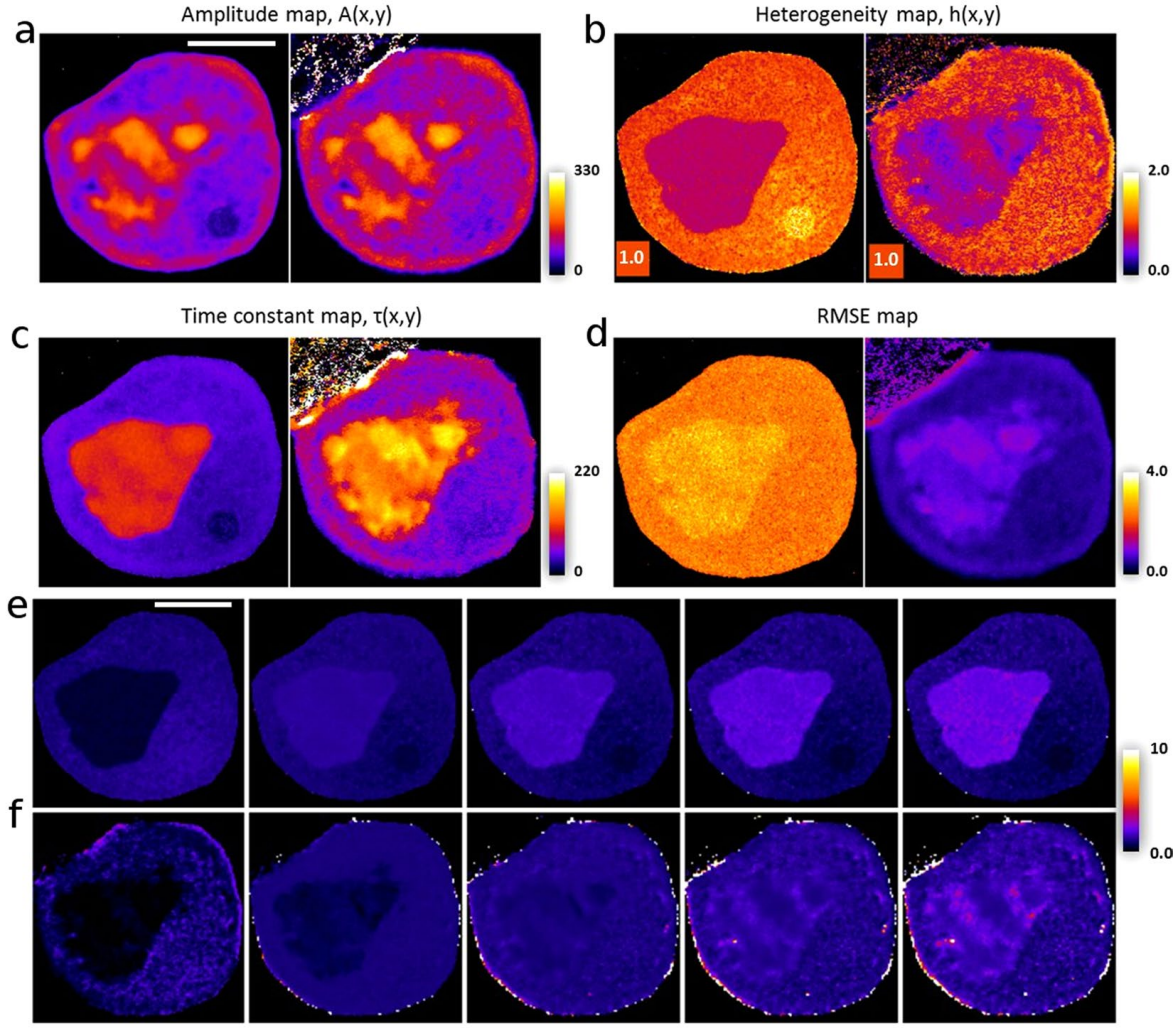
Pixel-wise comparison of temporal evolution of fluorescence loss between experiment and model (**a**–**d**); Pixel-wise fitting of a StrExp function to the calibrated model FLIP data (left panels) or to the experimental FLIP sequence (right panels) for the amplitude maps (**a**), the heterogeneity maps (**b**), the time constant maps (**c**) or the RMSE maps (**d**). The range is indicated, and the rectangular inset in panel (b) color–codes h = 1, as a reference value for a mono-exponential decay. (**e**,**f**) Selected frames of the temporal profile of rate coefficients (16) for the calibrated (**e**) and experimental FLIP data (**f**). The scale bars in (**a**) and (**e**) are 5 *μ*m. See text for further explanations.

Here, *I*_*n*_(**x**, *t*) = exp(−(*t*/*τ*(**x**))^(1/*h*(**x**))^) refers to the intensity decay normalized to the initial fluorescence given an amplitude equal to one^17,52^. For a stretched decay, the rate coefficient decreases over time, while for a compressed decay, the rate coefficient increases, indicating respective slowing and accelerating fluorescence loss kinetics at a given position^17^. We fitted this function to the experimental and calibrated FLIP sequence using a plugin, which we presented previously to the popular image analysis program ImageJ^18^ named PixBleach^55,56^. As shown in Fig. 8, the outcome of the FLIP simulation and calibration coincides nicely with the experimental FLIP data including spatially heterogeneous amplitude and time constant maps. As for the experimental data, fluorescence loss in the nucleus is significantly slowed, and the nucleus shows spatially varying fluorescence loss kinetics in experiment and FLIP simulation. From that, we conclude that our model, using spatially varying binding/release rate constants can accurately describe the experimentally known heterogeneity of nuclear diffusion of GFP, even though, we do not explicitly model spatially varying diffusion (i.e., we kept D spatially invariant and varied local binding affinities to unknown subcellular structures)^17,23^. The spatially varying intensity of GFP is observed at steady state in living McArdle cells and has been reported in many other studies as well^23,57^. Local differences in diffusion of GFP have been measured by fluorescence correlation spectroscopy (FCS) in the nucleus of HeLa cells, ranging from *D* ≈ 10 *μ*m^2^/*s* to *D* ≈ 35 – 50 *μ*m^2^/*s*, but those differences in diffusion were not correlated with GFP intensity in the same regions^23^. It is likely that the compact nuclear DNA creates local barriers to diffusion^28^, which we detect as locally delayed fluorescence loss profiles^17^.

As long as diffusion barriers are penetrable for GFP on the time scale of its cellular turnover by synthesis and degradation no spatial gradients of this protein should be expected. In other words, barriers can cause protein confinement on a short time scale but should lead to normal diffusion on a long time scale and therefore to a complete exploration of the three-dimensional nuclear space. As a consequence, any concentration gradients will be smoothened out and a homogeneous nuclear intensity of GFP would be expected. If on the other hand, the affinity of GFP for various nuclear subregions varies, a heterogeneous steady state distribution can be expected. Coexisting phases due to differences in polyelectrolyte concentration and properties have been proposed to contribute to the nuclear organization^31^, and GFP could show different affinities for such nuclear domains. Thus, our simplified mass-action model, while ignoring intradomain diffusion, emphasizes exchange of GFP between nuclear areas of different affinities for this protein. The same is true, though to a lower extent, for the cytoplasm. Similarly, the nuclear membrane can be seen as a barrier to diffusion, detectable by a variant of FCS^58^. The time constant map inferred from fitting the StrExp function to the experimental FLIP sequence or to the calibrated FLIP model data changes abruptly at the nuclear membrane, demonstrating that our computational FLIP model can detect barriers to diffusion as well (Fig. 8c). Also, the heterogeneity map and the maps of rate coefficients indicate delayed fluorescence loss in the nucleus for the experimental and calibrated FLIP sequence (compare Fig. 8b and e,f). This delay, characterized by a compressed StrExp function with increasing rate coefficients as function of time is a direct consequence of the presence of two effects: i) the nuclear membrane, acting as stringent barrier to diffusion and ii) hindrance to diffusion combined with partitioning preference of GFP in domains in the nucleus, which also causes the higher overall accumulation of GFP in that compartment compared to the cytoplasm. Both, the comparable shape of the fluorescence loss kinetics and the nuclear accumulation of GFP despite passive permeation across the nuclear membrane, are important validations of our reaction-diffusion FLIP model. Interestingly, on a smaller spatial scale (i.e., in the range of a few microns) the heterogeneity map is more structured for the experimental FLIP data than for the calibrated model (Fig. 8b). This leads to a larger spatial variation of the bleaching rate coefficients in the experimental FLIP sequence compared to the FLIP model (compare Fig. 8e and f, especially in the nucleus). It is likely that this minor discrepancy is a result of anomalous diffusion processes, which are not taken into account in our model^59^.

For further validating our model of passive permeation across the nuclear membrane, we made use of the data by Mohr *et al*.^24^, who compared the size dependence of nuclear permeation of various inert and spherical probe molecules^24^. The passive (i.e. not receptor mediated) influx of each studied molecular species followed first order kinetics, and the measured influx rate constant in permeabilized HeLa cells could be used to estimate the membrane permeability as p = k · V/A (nuclear volume, *V* = 1130 *μ*m^3^ and nuclear area, *A* = 540 *μ*m^2^). With these values and the Stokes-Einstein relation, we have performed a forward simulation of a FLIP experiment with selected probe molecules of very different Stokes radius (Supplemental Fig. S8). Clearly, increasing the Stokes radius from 0.67 nm for Fluorescein-tagged cysteine (Fl-Cys), over 1.69 nm for Ubiquitin (Ubq) to 2.85 nm for maltose-binding protein (MBP) had a dramatic effect on the fluorescence loss kinetics in the nucleus. While the nuclear membrane presented not much of a barrier for the nucleocytoplasmic exchange of Fl-Cys, permeation of MBP was strongly hindered. On the same time scale, lateral diffusion of all three probe molecules to the bleached area caused complete fluorescence loss in the cytoplasm (Supplemental Fig. S8). Together, these simulation results are in line with the experimental findings of Görlich and colleagues^24^, and shows the potential of our reaction-diffusion FLIP model to study nuclear transport and intracellular diffusion of other cargo molecules than GFP.

The simulation results of the calibrated FLIP model agree very well with the goal function and even the FLIP images in Fig. 6. The internal structure of the cell is accurately reproduced by the remarkably simple reaction–diffusion model. It might be worth noting that the FLIP images and hence also the goal function reflect a time interval of 1.8 s what it takes the confocal microscope to scan the image during the recovery phase after bleaching. The simulated images, however, display snapshots at discrete times *t* = 0, 26, 52 and 104 seconds.

By applying a discontinuous Galerkin method, it is possible to model the nuclear membrane as an internal interface instead of resolving the internal membrane dynamics as in^19^. As a consequence not only the DG mesh consists of 147 times fewer triangles, but also the PDE model is simpler replacing the internal membrane dynamics by the interface condition (8). The typical runtime for the simulation of a FLIP sequence is about 108 times faster than for the continuous Galerkin method. Also, this result exceeds the expectation formulated in the introduction. One reason is that the PDE model (7) consists of only two equations instead of four as in^19^.

In the literature, one can find several papers using a semipermeable membrane model, see^34,51,60^. Peters^51^ measures the permeability constant for a liver cell with a different size of dextrans. The article presents results for dextrans with a molecular mass of 19.5, 39.0 and 62.0 kDa. Although only three measurements are presented, it is clear that the correlation between the mass of the molecules and the respective measured permeabilities 0.705, 0.027 and 0.0036 *μ*m/s is nonlinear. As we only have three data points, a fit would be strongly biased by the error in the data. For GFP with its estimated Stokes radius of 2.42 nm and a molecular mass of 27 kDa however, one may expect a permeability in the lower range of the interval (0.027, 0.705). The estimated permeability for GFP of *p* = 0.111 clearly matches with that expectation.

It is also possible to model active transmembrane dynamics in the framework of a discontinuous Galerkin method. In that case, the semipermeable membrane condition (8) will be replaced by an active membrane condition based on reaction kinetics. A follow–up article is in preparation.

### Data availability

Experimental FLIP sequences, simulated images and program code will be made available by the authors upon request.

## Electronic supplementary material

Supplemental data
