# Supplementary material for: A Discontinuous Galerkin Model for Fluorescence Loss in Photobleaching
Source: Sci Rep. 2018 Jan 23;8:1387. doi: 10.1038/s41598-018-19159-7 (PMC5780497; doi:10.1038/s41598-018-19159-7)
Supplement: Supplementary file 1 — Supplemental data [file 41598_2018_19159_MOESM1_ESM.pdf]

A Discontinuous Galerkin Model for Fluorescence Loss in Photobleaching

Christian V. Hansen[1], **Hans J. Schroll**[2], **Daniel Wüstner**[3]

[1,2,3] *University of Southern Denmark, Campusvej 55, DK-5230 Odense M, Denmark*
[1] *Department of Mathematics and Computer Science* cvh@imada.sdu.dk
[2] *Department of Mathematics and Computer Science* achim@imada.sdu.dk
[3] *Department of Biochemistry and Molecular Biology* wuestner@bmb.sdu.dk

S Supplementary Information

S .1 Verification of the calibration algorithm

To verify the constrained optimization process, we perform a calibration based on synthetic data. The goal function is emulated by computing the FLIP model (7)-(10) on the mesh displayed in Figure 3 and with the parameters

$$\alpha = 20 \ , \quad \beta = 15 \ , \quad \gamma = 0.4 \ , \quad p = 0.1 \ . \tag{s.1}$$

When started at the initial guess

$$\alpha_0 = 25 \ , \quad \beta_0 = 20 \ , \quad \gamma_0 = 0.5 \ , \quad p_0 = 0.05 \ , \tag{s.2}$$

the Nelder-Mead algorithm detects the reference parameters (s.1) after 165 iteration steps with 185 evaluations of the misfit function to 5 digits precision and with a misfit functional $E = 0.00013$. Repeated calibrations from a variation of initial guesses confirm the robustness of the method.

S .2 Convergence test

To test convergence under mesh refinement, we consider the FLIP model (7)-(10) on a simplified, rectangular domain of size $10 \times 8\mu$m consisting of two compartments and a bleaching area within the right compartment as illustrated in Figure S1. The model parameters are (s.1) and the initial intensity is set to

$$c_0(x) = \sin(x/2) + 2 \ .$$

On a fine mesh consisting of 21150 triangles and with fixed time step $\Delta t = 0.2$ a reference solution is computed up to time $T = 13$. This reference solution is used to compute the L_2 error in space at final time on a sequence of coarser meshes and to estimate the order of convergence. We observe approximately linear convergence with respect to the maximal cell diameter $h_{\max}$ as presented in Table S1. A least square fit of the rates based on the five finest meshes gives an estimated order of convergence of $EOC = 1.02$. Given the fact that the approximate solution is discontinuous along the internal interface, the observed rates can be regarded as satisfying. Convergence of the method is clearly verified.

$h_{\max}$	triangles	est. error	EOC
1.4665	214	1.197796	-
0.9485	466	0.877973	0.71
0.8482	676	0.819151	0.62
0.7137	854	0.773946	0.32
0.5747	1294	0.673180	0.64
0.4425	2516	0.526906	0.94
0.3794	3408	0.472851	0.70
0.3010	5162	0.399167	0.84
0.2262	9752	0.265909	1.29
0.1983	12822	0.237004	0.87
0.1797	14986	0.219800	0.77

Table S1: Convergence test results.

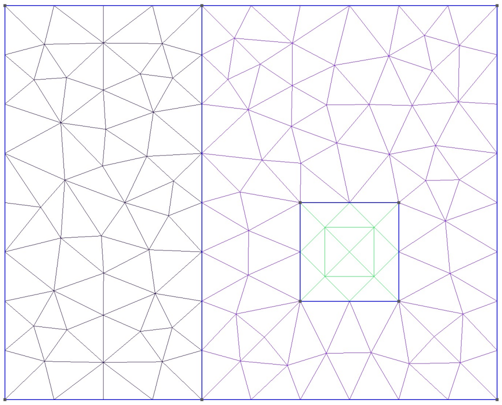

Figure S1: Rectangle mesh with 214 triangles. Left part emulates the nucleus, right part the cytoplasm containing the bleaching area (the square with green triangles).

S .3 Error analysis with respect to number of FLIP images

One of the natural questions that arise when doing FLIP imaging is, how long does the sequence need to be for reliable FLIP data modeling. Especially for the simulation and calibration cases the length of the FLIP sequence is important as each iterative time step can be very time-consuming. In this section, it is therefore tested how big impact the length of the FLIP sequence has on the precision of the calibration.

The calibration test is based on synthetic data. Thus the goal function is emulated by computing the PDE FLIP model on the 2D mesh displayed in Figure 3 and with the parameters

$$\alpha = 20 \ , \quad \beta = 15 \ , \quad \gamma = 0.4 \ , \quad p = 0.1 \ .$$

When started at the initial guess

$$\alpha_0 = 25 \ , \quad \beta_0 = 20 \ , \quad \gamma_0 = 0.5 \ , \quad p_0 = 0.05 \ .$$

Figure S2 presents 250 calibrations within 5 different groups. Each group represents the error measured on 50 independent calibrations with 25, 50, 75, 100, 125 comparisons to the goal function per iteration, respectively. The error calculated in each of the calibrations is found with respect to the same synthetic FLIP sequence with known parameters, for each calibration, there is added random Gaussian noise to the synthetic goal sequence. An error minimization with respect to four parameters can be very time consuming, especially for the case with 125 comparisons, as each iteration then consists of 1625 Euler steps, see Section 5. Thus, to reduce the complexity, the parameters β and γ are fixed, for the calibrations in Figure S2.

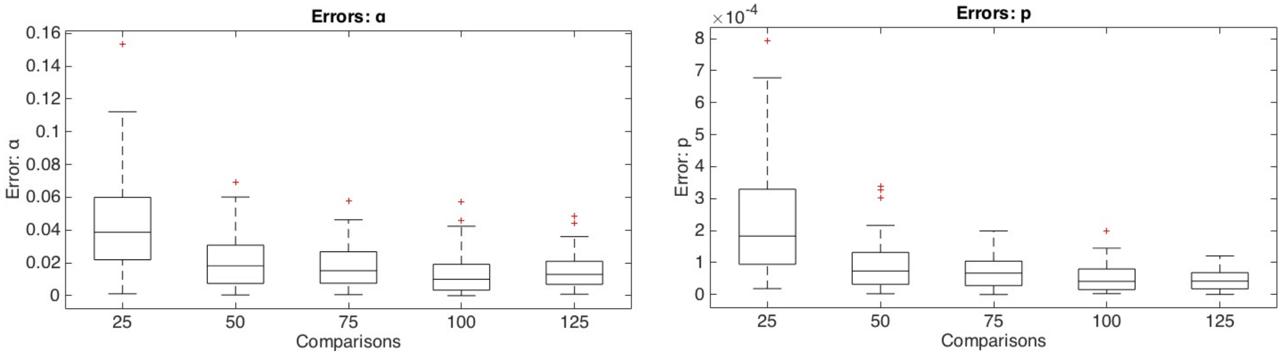

Figure S2: Boxplot of the errors for α and p, when β and γ is fixed, with different number of comparisons to the goal function.

Figure S2 shows that even though only 25 comparisons to the first 25 goal FLIP images are used, it is possible to achieve a quite actuate estimate of the parameters with respect to their size. Also, note that from 25 to 50 comparisons, the median of the error is reduced by half. However, increasing the number of comparisons to more than 50 would not give a significantly better result, especially not if compared to the increased computation time. Same experiment for calibration of β and γ, with α and p fixed is shown on Figure S3.

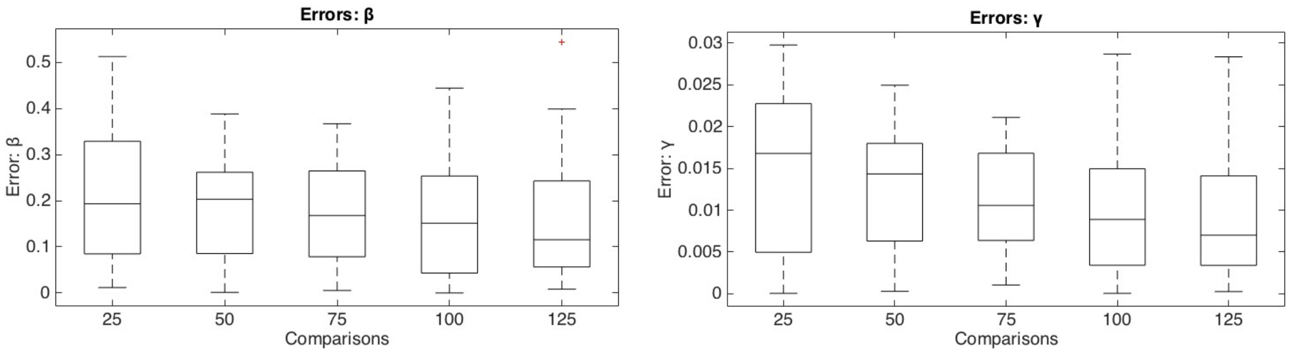

Figure S3: Boxplot of the errors for β and γ, when α and p is fixed, with different number of comparisons to the goal function.

The results in Figure S3 shows that the error might be reduced by increasing the number of comparisons, however, it doesn't show any significant improvement.

S .4 Error analysis with respect to the norm

The errors calculated in the previous section are given by the L_2-norm of the difference between the simulation and the goal function derived from the FLIP images. In Figure S4 test with the L_1, L_2 and H_1 norm are shown. As in Section S .3 each norm test consists of 50 calibrations with the synthetic FLIP sequence as goal function. The parameters β and γ are fixed, thus calibration is done with respect to α and p.

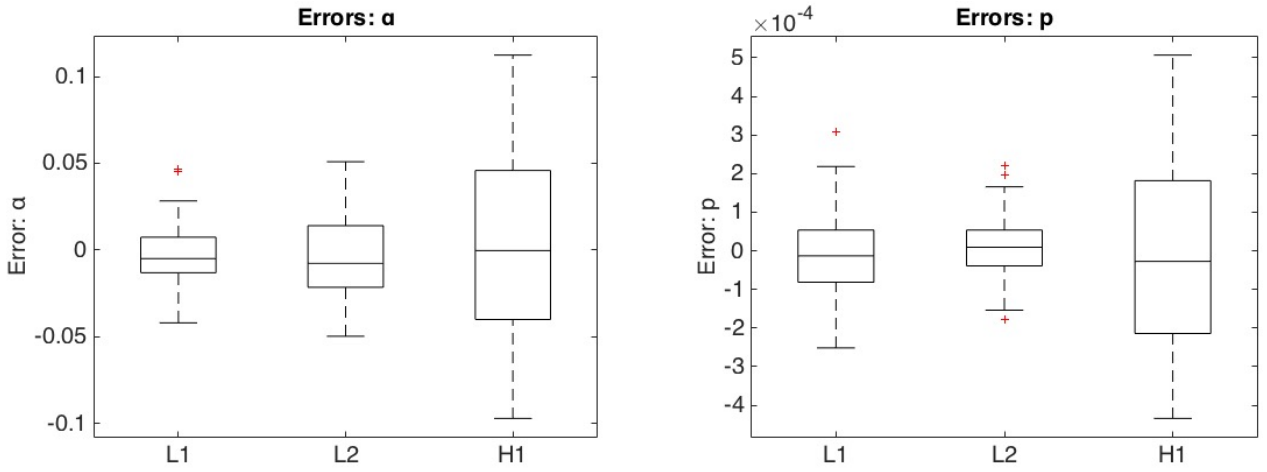

Figure S4: Boxplot of the errors for α and p, when β and γ is fixed, with different norms.

Figure S4 shows that the error obtained with the use of H_1-norm can be significantly larger than for the L_1 and L_2-norm. However, it is not possible to tell whether L_1 or L_2 is the better. The number of iterations for the calibration to converge is approximately the same for all the three norms. The L_2 and H_1-norm are build into the errornorm function in FEniCS and thus easier to use.

S .5 Optimization algorithms

Calibrating parameters by minimizing the error function is done by the use of the Nelder-Mead method. However, the choice of the best optimization algorithm is not trivial and strongly depends on the problem. In this section three different optimization algorithms are tested on our problem, the Broyden–Fletcher–Goldfarb–Shanno (BFGS) algorithm (see **SciPy**), the Limited-memory BFGS (L-BFGS) (see **Byrd et al.**; **Morales et al.**; **Zhu et al.**) and Nelder-mead (see **Nelder et al.**; **Sauer**). which are all a part of the **SciPy** library. First test shown in Figure S5 test the precision of the calibration of the parameters α and p, when β and γ are fixed. Each algorithm is tested 50 times, analog to the setup in Section S .3.

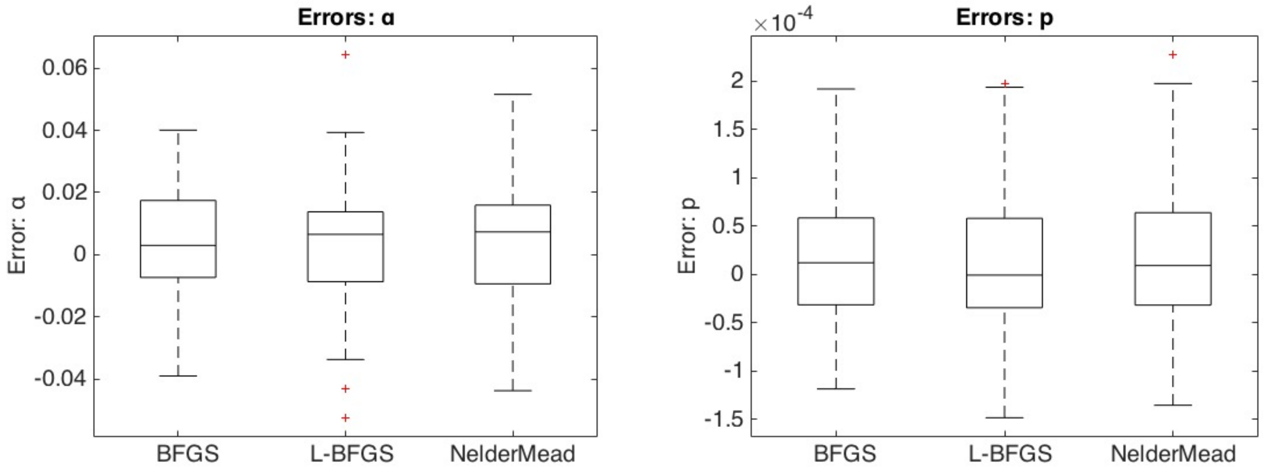

Figure S5: Boxplot of the errors for α and p, when β and γ is fixed, with different optimization algorithms

The different algorithms do of course have different stop criteria. For BFGS the iteration stops if a local minimizer is found and the gradient is small $\|g_k\|_\infty \leq 10^{-4}$. L-BFGS stops if either $\|g_k\|_\infty \leq 10^{-4}$ or the relative progress is small

$$\frac{(E^k - E^{k+1})}{\max(|E^k|, |E^{k+1}|, 1)} \leq 10^{-7} \; .$$

For Nelder-mead the iteration stops if either the absolute value of the largest stepsize between the found parameters is less than 10^{-3} or the absolute change in the error function between iterations is lower than 10^{-4}. These stop criteria are chosen for moderate accuracy (see **SciPy**; **Zhu et al.**). But despite the different stop criteria Figure S5 and Figure S6 shows that the three different optimization algorithms find the parameters with approximately same precision and same minimum for the calibration error.

Thus given the approximately same result, it is ideal to look at the computation time for each algorithm. The computation times for the calibration process can be found in Figure S7, which shows that the median value for the calibration process using the L-BFGS algorithm is approximately three times as slow as the fastest algorithm Nelder-Mead.

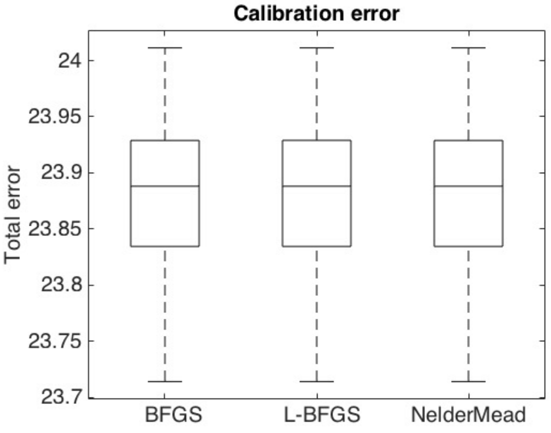

Figure S6: Calibration error for calibration of α and p with different algorithms.

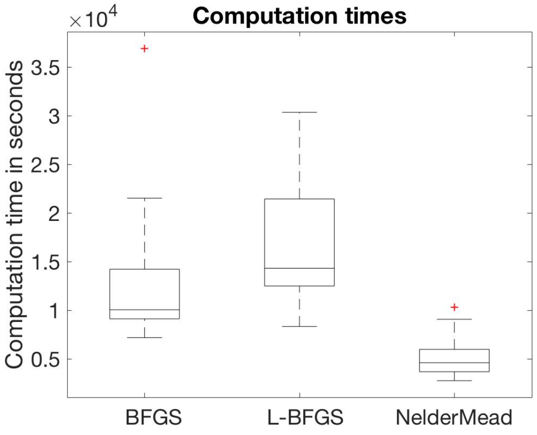

Figure S7: Computation times for calibration of α and p with different algorithms.

S .6 Bleaching sensitivity

For the FLIP experiment shown in Figure 6A-D, the diameter of the bleaching area is 30 pixels. To investigate the effect and sensitivity of the bleaching coefficient β and permeability p when varying the size of the bleaching area on the calibration mesh, we setup an experiment where α and γ are fixed and set to $\alpha = 16.1$ and $\gamma = 0.319$ from the estimates presented in (15).

Diameter	β	$\bar{p}$
20	106	0.111
30	35.6	0.111
40	15.3	0.111

Table S2: Sensitivity test with respect to the size of the bleaching area on the mesh used for calibration.

The results in Table S2 shows that the size of the bleaching area is inversely proportional to the bleaching parameter β, meaning that if one increases the size of the bleaching area in the mesh used for calibration, the calibrated bleaching parameter β will be lower. The change in the diameter of the bleaching area does not affect the permeability constant p. This is what one would expect, as the goal function for the calibration in all three experiments are the same, i.e., if one increased the bleaching area and did not change β, one would obtain a larger bleaching effect than the one seen in the goal function.

S .7 Molecular size

Figure S8 (C-N) shows simulation results for different sized molecules. All three simulations have the same initial values, k^+, k^- and we set $\beta = 35.6$ and $\gamma = 0.319$. The first four images (C-F) are from a simulation with permeability and diffusion like Fluorescein-Cys i.e. $p = 1.97$ and $\alpha = 84.52$. The left most cFLIP image (C) is from before bleaching, the next image (D) is after it has been bleached 10 times i.e. time $t = 26$ s. The third cFLIP image (E) is the 20'th cFLIP image in the sequence (time $t = 52$ s) and the last (F) is at time $t = 104$ s which correspond to cFLIP fame 40. The second row (G-J) shows a simulation with permeability and diffusion like Ubiquitin i.e. $p = 0.07450$ and $\alpha = 33.51$. The third row (K-N) shows a simulation of MBP with $p = 0.00046$ and $\alpha = 19.87$. See the Discussion section in the paper for further details.

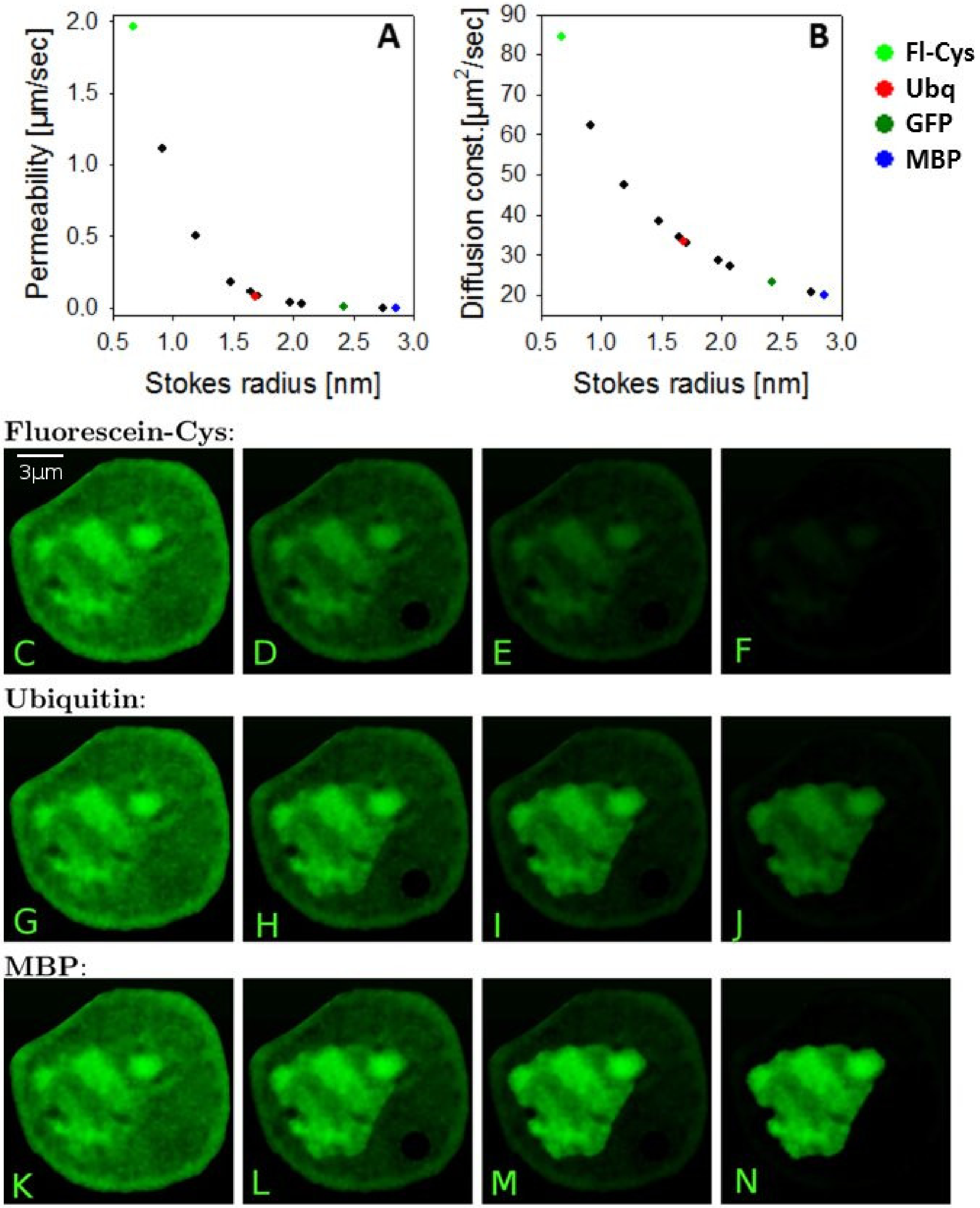

Figure S8: A, B. Simulation of permeability (A) and 2D-diffusion of various inert probe molecules for passive permeation across the nuclear membrane according to the data of **Mohr et al.** Probe molecules selected for FLIP simulation are fluorescein-tagged cysteine (Fl-Cys, light green symbols), ubiquitin (Ubq, red symbols), GFP (dark green symbols) and maltose binding protein (MBP, blue symbols). C-N, selected frames from a forward FLIP simulation using the permeability values and diffusion constants shown in A, B for Fl-Cys (C-F), Ubq (G-J) and MBP (K-N).

References

[1] Byrd R. H., Lu P. and Nocedal J.: A Limited Memory Algorithm for Bound Constrained Optimization. *SIAM Journal on Scientific and Statistical Computing, 16, 5, pp. 1190-1208.* (1995)

[2] Mohr D., Frey S., Fischer T., Guttler T. and Gorlich D.: Characterisation of the passive permeability barrier of nuclear pore complexes. *The EMBO journal 28: 2541-2553.* (2009)

[3] Morales J.L. and Nocedal J.: Remark on "Algorithm 778: L-BFGS-B, FORTRAN routines for large scale bound constrained optimization". *ACM Trans. Math. Softw., 38, pp. 7:1-7:4* (2011)

[4] Nelder J.A. and Mead R.: A simplex method for function minimization. *The Computer Journal, 7, 308-313.* (1965)

[5] Sauer T.: Numerical Analysis. *Pearson Education* (2006)

[6] Scientific Computing Tools for Python: SciPy `http://scipy.org`

[7] Zhu C., Byrd R. H. and Nocedal J.: Algorithm 778: L-BFGS-B, FORTRAN routines for large scale bound constrained optimization. *ACM Trans. Math. Softw., 23, 4, pp. 550-560.* (1997)
